# Detecting cortical circuits resonant to high-frequency oscillations in the human primary motor cortex: a TMS-tACS study

**DOI:** 10.1038/s41598-020-64717-7

**Published:** 2020-05-06

**Authors:** Andrea Guerra, Federico Ranieri, Emma Falato, Gabriella Musumeci, Alessandro Di Santo, Francesco Asci, Giovanni Di Pino, Antonio Suppa, Alfredo Berardelli, Vincenzo Di Lazzaro

**Affiliations:** 1 IRCCS Neuromed, Via Atinense 18, 86077 Pozzilli, (IS) Italy; 2 Department of Neuroscience, Biomedicine and Movement Sciences, University of Verona, P.le L.A. Scuro 10, 37134 Verona, Italy; 3 Unit of Neurology, Neurophysiology, Neurobiology, Department of Medicine, University Campus Bio-Medico, via Álvaro del Portillo 21, 00128 Rome, Italy; 4 Department of Human Neurosciences, Sapienza University of Rome, Viale dell’Università 30, 00185 Rome, Italy; 5 Unit of Neurophysiology and Neuroengineering of Human-Technology Interaction, Department of Medicine, University Campus Bio-Medico, via Álvaro del Portillo 21, 00128 Rome, Italy

**Keywords:** Motor cortex, Neural circuits, Neuronal physiology

## Abstract

Corticospinal volleys evoked by transcranial magnetic stimulation (TMS) over the primary motor cortex (M1) consist of high-frequency bursts (≈667 and ≈333 Hz). However, intracortical circuits producing such corticospinal high-frequency bursts are unknown. We here investigated whether neurons activated by single TMS pulses over M1 are resonant to high-frequency oscillations, using a combined transcranial alternating current stimulation (tACS)-TMS approach. We applied 667, 333 Hz or sham-tACS and, concurrently, we delivered six single-pulse TMS protocols using monophasic or biphasic pulses, different stimulation intensities, muscular states, types and orientations of coils. We recorded motor evoked potentials (MEPs) before, during and after tACS. 333 Hz tACS facilitated MEPs evoked by biphasic TMS through a figure-of-eight coil at active motor threshold (AMT), and by monophasic TMS with anterior-to-posterior-induced current in the brain. 333 Hz tACS also facilitated MEPs evoked by monophasic TMS through a circular coil at AMT, an effect that weakly persisted after the stimulation. 667 Hz tACS had no effects. 333 Hz, but not 667 Hz, tACS may have reinforced the synchronization of specific neurons to high-frequency oscillations enhancing this activity, and facilitating MEPs. Our findings suggest that different bursting modes of corticospinal neurons are produced by separate circuits with different oscillatory properties.

## Introduction

**Figure 1:**
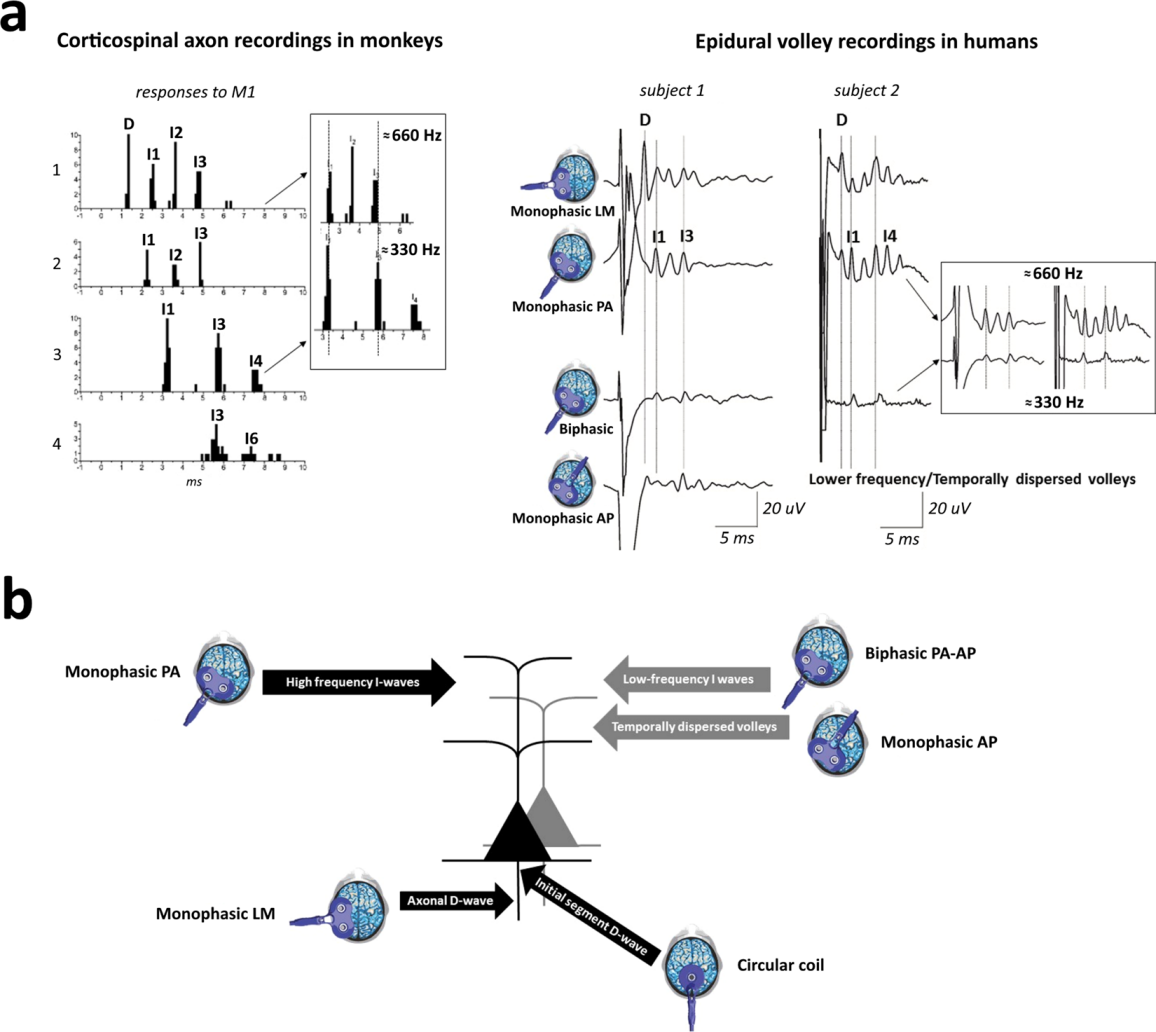
Cortical circuits activated by motor cortex simulation. *Panel a. Left:* Post stimulus time histograms of individual axon responses after motor cortex (M1) intracortical stimulation in monkeys (modified from^6^). The peaks of the histograms reveal different patterns of discharge: 1. high-frequency (≈660 Hz) repetitive discharge with a D-wave (i.e. the earliest response originating from the direct activation of corticospinal axons) followed by several I-waves (i.e. longer latency responses originating from indirect activation of corticospinal cells through trans-synaptic inputs); 2. high-frequency repetitive discharge with only I-waves; 3. lower frequency discharge at ≈330 Hz (see the insert); 4. more temporally-dispersed responses. *Right:* Corticospinal volleys recorded at epidural level after different forms of magnetic stimulation in two human subjects. Monophasic lateromedial (LM) TMS evokes a high-frequency (≈660 Hz) repetitive discharge with a D-wave followed by several I-waves, monophasic posterior-to-anterior (PA) TMS preferentially evokes only I-waves, biphasic TMS evokes a lower frequency activity (≈330 Hz, see the insert for subject 2), monophasic anterior-to-posterior (AP) TMS evokes both lower frequency and temporally dispersed responses. *Panel b*. Diagrammatic representation of possible sites of activation of corticospinal neurons (CSNs) using different TMS techniques on M1. Two pyramidal tract neurons are represented. Monophasic PA TMS preferentially activates CSNs trans-synaptically evoking high-frequency I-waves; biphasic PA-AP TMS and monophasic AP TMS preferentially activate trans-synaptically different CSNs, evoking lower frequency I-waves and temporally dispersed volleys; TMS with a circular coil centred over the vertex preferentially activates CSNs at the level of the axon hillock; monophasic LM TMS preferentially activate the corticospinal axons at some distance from the cell body.

Corticospinal neurons (CSNs) of the mammalian brain show a high frequency (≈667 Hz) burst of activity in response to transcranial electric (TES) and magnetic (TMS) stimulation. These stereotyped bursts of activity can be recorded from the surface of the high cervical cord and reflect the spiking of a large number of corticospinal axons^1–5^. Recently, Maier *et al*.^6^ recorded the responses of single corticospinal axons together with volleys from the surface of the cervical cord after intracortical stimulation in monkey and showed that individual axons fire repetitively at the high frequency revealed by surface recordings, thus demonstrating that bursts originate from the repetitive synchronous discharge of CSNs. They also found that while most of the corticospinal axons discharged at around 600 Hz, there were other axons responding at lower frequencies^6^. In humans, bursts of corticospinal activity with different frequencies can be recorded by cervical epidural electrodes after TMS over the motor cortex^5^. This descending bursting activity is influenced by the direction of the current flowing across the central sulcus. The more commonly used posterior-to-anterior (PA)-induced current in the brain (perpendicular to the central sulcus) preferentially evokes the 667 Hz repetitive discharge. However, when the orientation of the induced current is reversed (anterior-to-posterior in the brain; AP) or when stimulation is performed using a biphasic TMS that combines sequentially both directions of stimulation (a PA induced current followed by an AP induced current), the output changes with less synchronized volleys with peak latencies later than those seen after PA stimulation. The corticospinal output evoked by AP and biphasic stimulation is more variable but, in a few subjects, bursts of activity with a frequency that is half of that of the PA-evoked bursts (≈333 Hz) were recorded^5^. Thus, both animal and human data suggest that multiple cortical circuits can be activated by motor cortex stimulation, producing different frequencies of discharge of CSNs (Fig. 1, see^5^ for a review). The physiological mechanisms leading to a lower frequency bursting of CSNs after AP and biphasic stimulation (when compared with PA stimulation) are still unclear. However, modelling studies suggest that PA stimulation preferentially activates the CSNs monosynaptically, producing highly synchronized corticospinal activity and MEPs with shorter latencies, while AP stimulation could activate CSNs polysynaptically, producing less synchronized corticospinal activity and MEPs with longer latencies^7^. However, to record the lower frequency descending activity the intensity of the stimulus is critical. Indeed, at higher stimulus intensities the lower frequency bursting is no longer evident, and it is replaced by the 667 Hz activity.

To date, it is unknown whether the increase of the synchronization of motor cortex circuits at 667 Hz and/or at the lower frequencies corresponding to the bursts evoked by AP or biphasic stimulation, would eventually result in an increase of the corticospinal output. Also, it is unclear whether corticospinal bursts at higher and lower frequencies are produced by independent oscillatory intracortical circuits with different characteristics, as suggested by modeling studies^8^, or rather reflect the activity of a single neuronal generator able to discharge at harmonically-related frequencies (667 and 333 Hz). A possible approach to explore this issue is to take advantage of the combined stimulation of the motor cortex with TMS and transcranial alternating current stimulation (tACS)^9–16^. tACS is able to cause coherent changes in the firing probability and thereby timing of neuronal populations, thus entraining brain activity^17–19^. Since this effect preferentially occurs when the stimulation frequency matches with the endogenous rhythm of the neurons being stimulated (‘resonance principle’^20–22^), tACS can be used to test the ability of brain areas, networks or neuronal elements to resonate at specific frequencies.

We here hypothesize that tACS at 667 Hz (the frequency that coincides with that of the most consistent and stereotyped activity evoked by TMS) and 333 Hz (the most consistent lower frequency observed in few human recordings) could differentially influence the response of motor cortex to TMS. Thus, we applied tACS over motor cortex at 667 and 333 Hz and, at the same time, we delivered TMS by using single monophasic or biphasic pulses, at different intensities, with different types of coils and coil handle orientations, so as to test as selectively as possible the excitability of circuits evoking the two bursting modes^5^. In order to test whether any significant effect was frequency-specific, we performed a control experiment using transcranial random noise stimulation (tRNS), a form of stimulation that includes a wide spectrum of frequencies^23^. The amplitude of motor evoked potentials (MEPs) produced by the different protocols of stimulation in hand muscles was the readout used to evaluate the effects of cortical circuit entrainment. Evaluation of amplitude of MEPs evoked by monophasic and biphasic stimulation can provide useful insights into the physiology of different sets of interneurons projecting to CSNs^24^. We posit that MEP amplitude increase might reflect the strengthening of the activity of neural elements in M1 due to the reinforced synchronization of intracortical circuits at high frequencies through tACS. Finally, in order to verify whether such putative effects only occur during tACS or, also outlast the stimulation, we also repeated the same recordings after tACS.

## Methods

### Participants

Thirty healthy right-handed subjects (17 males; mean age ± SD: 26.6 ± 3.8 years) were enrolled in the study. None of the participants had any neurological or psychiatric disorders, and none were taking drugs known to modulate brain excitability. No participant had any contraindication to TMS or transcranial electrical stimulation, as indicated in the current international safety guidelines^25,26^. The study was conducted in accordance with the Declaration of Helsinki and approved by the Ethics Committee of University Campus Bio-Medico of Rome. All subjects gave their written informed consent for participating in the study.

### tACS and tRNS

tACS and tRNS were performed by using two conductive rubber electrodes enclosed in sponges soaked in saline solution (size: 5 × 7 cm) through a BrainSTIM stimulator (EMS, Bologna, Italy). The stimulating electrodes were centered over the dominant (left) M1 and over the Pz point of the 10–20 EEG system respectively, similarly to our previous studies^11–13,27,28^. Electrodes were secured in place by using elastic bands. Impedance was kept at <10 kΩ, as measured by the stimulation device. tACS was delivered at two different frequencies: 667 Hz and 333 Hz. In addition, sham stimulation was used as a control condition, consisting in 667 Hz tACS activated for only 7 seconds before applying TMS. Sinewave stimulation was delivered with no direct current offset and a peak-to-peak amplitude of 1 mA with 3-second ramping-up and ramping-down periods. This intensity did not induce visual or skin sensations in any participant. Accordingly, no subject was able to distinguish among the different stimulation conditions. Other than in the sham condition, tACS was kept active for the whole duration of TMS protocols (i.e. 8–9 mins, see below). tRNS was delivered in a range unbalanced toward the high-frequencies (10–640 Hz), and the stimulation intensity was set at 1 mA.

### TMS

TMS was carried out by means of a Magstim 200^2^ stimulator, delivering monophasic pulses, and a Magstim SuperRapid stimulator, delivering biphasic pulses (Magstim Co Ltd, Whitland, South West Wales, UK). We used a standard figure-of-eight 70 mm coil (Magstim Co Ltd) (focal coil) or a large circular 90 mm coil (Magstim Co Ltd) (circular coil) according to the specific protocol used. MEPs were recorded from the right first dorsal interosseous (FDI) muscle of the hand. The ‘hotspot’ (i.e. optimal scalp position to elicit MEPs) of the FDI muscle was identified with the handle of the TMS coil pointing posteriorly and laterally or anteriorly and medially to the midsagittal line, depending on the protocol used (see below). This procedure was repeated twice: first, in order to center the stimulating electrode of tACS over the dominant (left) M1; second, after the electrodes had been positioned on the participant’s head, when the site was marked over the sponge in order to ensure reliable coil repositioning during the experiment. Resting motor threshold (rMT) and active motor threshold (AMT) were then determined according to the international guidelines^29^. RMT was defined as the minimum stimulation intensity able to elicit MEPs (at least 50 μV in amplitude) in 50% of 10 consecutive stimuli. AMT was determined during a mild tonic contraction of the FDI muscle (approximately 20% of the maximal muscle strength) and defined as the minimum stimulation intensity eliciting MEPs of about 200 μV in amplitude in 50% of 10 consecutive stimuli.

Six different single-pulse TMS protocols were performed, according to the motor state of the participant, the magnetic pulse type, the coil shape and orientation used:

1. *rMT-mono*: monophasic TMS pulses delivered at rest through a focal coil with PA-induced current (posterior-to-anterior handle orientation), at the intensity of 100% rMT;
2. *110 rMT-mono*: monophasic TMS pulses delivered at rest through a focal coil with PA-induced current, at the intensity of 110% rMT;
3. *AMT-mono*: monophasic TMS pulses delivered during tonic contraction of the FDI muscle (about 20% of maximal voluntary muscle contraction) through a focal coil with PA-induced current, at the intensity of 100% AMT;
4. *AMT-biph*: biphasic TMS pulses delivered during voluntary muscular contraction through a focal coil with PA-AP-induced current, at the intensity of 100% AMT;
5. *AMT-circ*: monophasic TMS pulses delivered during voluntary muscular contraction through a circular coil with an anticlockwise current flow, at the intensity of 100% AMT;
6. *AMT-mono AP*: monophasic TMS pulses delivered during voluntary muscular contraction through a focal coil with AP-induced current (anterior-to-posterior handle orientation), at the intensity of 100% AMT.

In protocols 1–4 and 6 the TMS coil was oriented so as to induce a current flow approximately perpendicular to the central sulcus (PA for protocols 1–3, PA-AP for protocol 4, and AP for protocol 6), whereas in protocol 5 the coil was centered over the vertex (Cz site of the 10–20 EEG system) with the handle pointing backwards. Protocols 1–3 preferentially activate the cortical circuits evoking CSN bursting at 667 Hz with trains of responses of longer duration at increasing stimulus intensities^5,30^. The use of three different levels of stimulation enhances the possibility to detect even subtle changes in the bursting activity. Protocol 4 and 6 preferentially evoke the 333 Hz bursting activity, whereas protocol 5 activates the CSNs at the level of the axon hillock, with a less pronounced activation of presynaptic inputs, thus providing more direct information on the level of excitability of these cells^5,30^. AMT-biph, AMT-circ and AMT-mono AP protocols were performed only during voluntary activity and at low intensity (AMT) because their selectivity for the circuit inducing lower frequency bursting (biphasic and AP stimulation) or for direct activation of CSNs (circular coil) has been demonstrated only at low stimulus intensities^5,30^ (Fig. 1). In protocols 3–6 the level of muscular contraction was visually monitored online by one of the researchers. In case the amount of contraction changed significantly during the course of the experiment, an auditory feedback was given to participants, so as to adjust their muscular activity and keep it constant during the recordings. MEPs were recorded through a pair of surface electrodes in a belly/tendon montage. Electromyographic (EMG) signals were amplified (Digitimer D360 amplifier; Digitimer Ltd, Welwyn Garden City, UK), digitized at 5 kHz (CED 1401 A/D converter; Cambridge Electronic Design Ltd, Cambridge, UK) and stored on a computer for off-line analysis (Signal software, Cambridge Electronic Design).

### Experimental design

**Figure 2:**
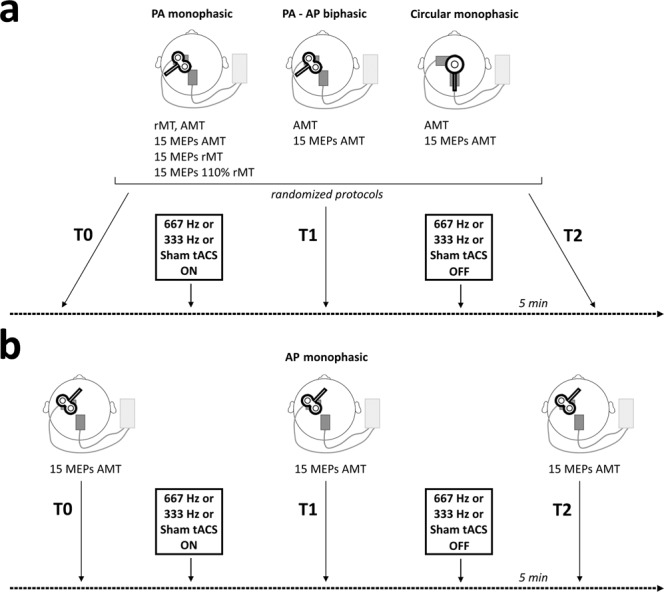
Experimental design. *Panel a*. In Experiment 1, five different TMS protocols were applied in a randomized order before activating tACS (T0), during tACS (T1) and 5 minutes after the end of tACS (T2) over M1: 1) monophasic TMS pulses delivered at rest through a standard figure-of-eight coil, at the intensity of 100% rMT; 2) monophasic TMS pulses delivered at rest through a standard figure-of-eight coil, at the intensity of 110% rMT; 3) monophasic TMS pulses delivered during mild voluntary muscular contraction through a standard figure-of-eight coil, at the intensity of 100% AMT; 4) biphasic TMS pulses delivered during mild voluntary muscular contraction through a standard figure-of-eight coil, at the intensity of 100% AMT; 5) monophasic TMS pulses delivered during mild voluntary muscular contraction through a circular coil, at the intensity of 100% AMT. *Panel b*. In Experiment 2, monophasic TMS pulses were delivered during mild voluntary muscular contraction through a standard figure-of-eight coil with an anterior-to-posterior handle orientation at T0, T1 and T2. In both experiments, each participant underwent three randomized sessions in which tACS was delivered at 667 Hz or 333 Hz frequency, or the stimulation was sham. PA = posterior-to-anterior; AP = anterior-to-posterior; AMT = active motor threshold; rMT = resting motor threshold.

Two main experiments have been performed in this study. In Experiment 1 (Fig. 2, panel a), fifteen participants (9 males; mean age ± SD: 25.5 ± 4.4 years) underwent three randomized experimental sessions, conducted at the same time of the day, at least one week apart: (i) 667 Hz tACS; (ii) 333 Hz tACS; (iii) sham tACS. Subjects were seated in a comfortable chair with their arms fully relaxed in a natural position and their hands resting on a table. After having positioned tACS electrodes over the scalp, rMT and AMT were determined for each TMS protocol. Then, 15 MEPs evoked by single-pulse TMS (4.5–5.5 seconds inter-stimulus interval) were recorded for each of the protocols 1–5 at three different time-points: before activating tACS (T0), during tACS (T1) and 5 minutes after having switched-off tACS (T2). The five different TMS protocols were tested consecutively, and their order was randomized at all the time-points. At T1, TMS was started ≈10 seconds after the activation of tACS. In Experiment 2 (Fig. 2, panel b), in a different group of fifteen participants (8 males; mean age ± SD: 27.7 ± 2.7 years), we tested the effect of 667 Hz, 333 Hz and sham tACS (randomly delivered in different sessions) on MEPs evoked by single-pulse TMS with the coil handle oriented in the AP direction (protocol 6). Similar to Experiment 1, 15 MEPs were recorded at T0, T1 and T2.

Finally, in a control experiment, the same fifteen participants of Experiment 1 underwent tRNS. MEPs evoked by single-pulse TMS were recorded for protocols 4 and 5 before, during and 5 minutes after tRNS.

### Data and statistical analysis

Peak-to-peak MEP amplitudes were measured by means of a customized script on Matlab software (The MathWorks Inc) and then averaged for each condition. In protocols performed at rest, trials displaying EMG activity >0.1 mV preceding TMS were discarded. Separate one-way repeated-measures (rm) ANOVAs with the factor ‘session’ (3 levels: 667 Hz tACS, 333 Hz tACS, sham tACS) were used to compare the rMT, AMTs (AMT-mono, AMT-biph and AMT-circ) and the amplitude of MEPs measured at T0 in the three experimental sessions. A three-way rmANOVA with ‘session’, ‘protocol’ (5 levels: rMT-mono, 110 rMT-mono, AMT-mono, AMT-biph, AMT-circ) and ‘time-point’ (3 levels: T0, T1, T2) as factors was used to test possible effects of tACS stimulation on MEPs amplitude in Experiment 1. In order to check whether the level of voluntary muscular contraction influenced our results, a rmANOVA with ‘protocol’ (3 levels: AMT-mono, AMT-biph, AMT-circ) and ‘time-point’ as factors was conducted on the mean rectified EMG signal amplitude, measured in the 100 ms preceding TMS. A two-way rmANOVA with ‘session’ and ‘time-point’ as factors was adopted to test possible changes of MEPs amplitude in Experiment 2. Finally, two separate rmANOVAs with ‘time-point’ as factor were used to verify possible effects of tRNS in the control experiment. Greenhouse-Geisser corrections were applied when a violation of sphericity was detected. The level of significance was set at p ≤ 0.05. Tukey’s honest significance test was subsequently applied for post-hoc comparisons. Unless otherwise stated, all the values are presented as mean ± standard error of means (SEM). Statistical analyses were performed using Statistica (StatSoft Inc).

The sample size was computed with desired power of 0.80 and alpha error of 0.05, assuming a 25% change in MEP amplitude from a baseline value of 1.0 ± 0.3 mV, based on previously published data on high-frequency tACS^31^. The minimal required sample size was calculated to be 12 subjects.

## Results

The RMT (F_2,28_ = 0.41, p = 0.67) and the AMTs (AMT-mono: F_2,28_ = 1.46, p = 0.25; AMT-biph: F_2,28_ = 0.18, p = 0.83; AMT-circ: F_2,28_ = 0.89, p = 0.42) were similar in the three different experimental sessions (Table 1). The amplitude of MEPs recorded at T0 was also comparable between sessions (rMT-mono: F_2,28_ = 0.82, p = 0.45; 110 rMT-mono: F_2,28_ = 0.001, p = 0.99; AMT-mono: F_2,28_ = 1.22, p = 0.31; AMT-biph: F_2,28_ = 1.50, p = 0.24; AMT-circ: F_2,28_ = 0.74, p = 0.49).

**Table 1:** Motor thresholds.

	AMT-mono (%)			AMT-biph (%)			AMT-circ (%)			rMT-mono (%)		
	667 Hz	333 Hz	Sham	667 Hz	333 Hz	Sham	667 Hz	333 Hz	Sham	667 Hz	333 Hz	Sham
*mean*	35.5	36.7	33.7	49.0	50.4	49.2	34.5	37.9	35.7	54.8	53.5	53.3
*SD*	6.0	9.1	8.4	13.7	13.3	14.3	8.5	9.9	5.9	9.8	11.2	12.3

Active motor thresholds (AMT-mono = AMT measured with monophasic TMS and figure-of-eight coil, AMT-biph = AMT with biphasic TMS and figure-of-eight coil, AMT-circ = AMT with monophasic TMS and circular coil) and resting motor threshold (rMT-mono = rMT measured with monophasic TMS and figure-of-eight coil) for each experimental session (mean and standard deviation - SD - values).

**Figure 3:**
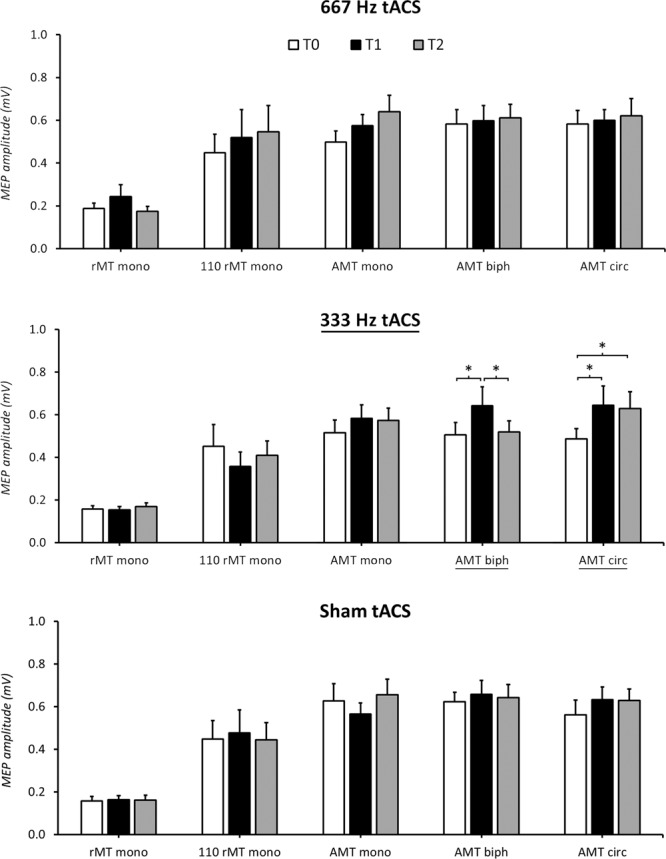
Effects of 667 Hz tACS (upper line), 333 Hz tACS (middle line) and sham tACS (lower line) on MEPs amplitude in the five different TMS paradigms tested. 333 Hz tACS facilitated MEPs in AMT-biph and AMT-circ protocols. Note that the tACS-induced increase of MEPs amplitude in AMT-biph was present only during the stimulation (T1), while in AMT-circ this effect persisted at 5 minutes after tACS (T2). 667 Hz tACS and sham tACS did not induce any significant effect on MEPs. Asterisks denote significant differences in the post-hoc analyses.

The rmANOVA conducted on MEP amplitude in Experiment 1 disclosed a significant ‘session’ × ‘protocol’ × ‘time-point’ interaction (F_16,224_ = 1.97, p = 0.016), suggesting that this measure was modified by tACS in one or more TMS protocols, during and/or after the stimulation. Also, a significant effect of the factor ‘protocol’ (F_4,56_ = 22.07, p < 0.001) was present, indicating that MEP amplitude was different according to the TMS protocol applied. As expected, post-hoc analysis demonstrated a smaller MEP size for rMT-mono with respect to all the other protocols (p < 0.001 for all the comparisons). The factors ‘session’ (F_2,28_ = 0.52, p = 0.60) and ‘time-point’ (F_2,28_ = 2.89, p = 0.07), as well as the ‘session’ × ‘protocol’ (F_8,112_ = 0.61, p = 0.77), ‘session’ × ‘time-point’ (F_4,56_ = 0.94, p = 0.45) and ‘protocol’ × ‘time-point’ (F_8,112_ = 1.73, p = 0.10) interactions were not significant. Then, three separate rmANOVAs with ‘protocol’ and ‘time-point’ as factors were conducted to identify which tACS frequency modulated MEPs. Both the 667 Hz tACS and the sham tACS left MEPs unchanged in all protocols, as demonstrated by the lack of a ‘protocol’ × ‘time-point’ interaction (667 Hz tACS: F_8,112_ = 1.01, p = 0.44; sham tACS: F_8,112_ = 1.35, p = 0.23) and the non-significant factor ‘time-point’ (667 Hz tACS: F_2,28_ = 2.21, p = 0.13 sham tACS: F_2,28_ = 0.62, p = 0.54). By contrast, when considering the 333 Hz tACS session, the rmANOVA disclosed a ‘protocol’ × ‘time-point’ interaction (F_8,112_ = 3.31, p = 0.002) and a significant factor ‘time-point’ (F_2,28_ = 3.67, p = 0.04). Separate follow-up rmANOVAs with the factor ‘time-point’ suggested that 333 Hz tACS modulated AMT-biph (F_2,28_ = 5.40, p = 0.01) and AMT-circ (F_2,28_ = 4.25, p = 0.02), but not AMT-mono (F_2,28_ = 1.96, p = 0.16), rMT-mono (F_2,28_ = 0.41, p = 0.66) and 110 rMT-mono (F_2,28_ = 1.72, p = 0.19). Post-hoc analysis indicated that in 333 Hz AMT-biph, MEPs were significantly facilitated at T1 (T0 vs T1: p = 0.01) and returned to their baseline amplitude at T2 (T0 vs T2: p = 0.96; T1 vs T2: p = 0.03). Differently, in 333 Hz AMT-circ, MEP amplitude was higher both at T1 (p = 0.04) and, to a lesser extent, at T2 (p = 0.05) than at T0. MEPs facilitation was comparable at T1 and T2 (p = 0.99) (Fig. 3). Since the modulation we found with 333 Hz tACS was present in protocols implying voluntary muscular contraction during TMS, we verified that the amount of EMG activity preceding TMS was not significantly different in all the time-points and protocols tested. The rmANOVA demonstrated no effect of the factors ‘time-point’ (F_2,28_ = 1.44, p = 0.25) and ‘protocol’ (F_2,28_ = 3.14, p = 0.06), and the lack of a ‘time-point’ × ‘protocol’ interaction (F_4,56_ = 0.19, p = 0.94), confirming similar EMG values throughout the experiment.

**Figure 4:**
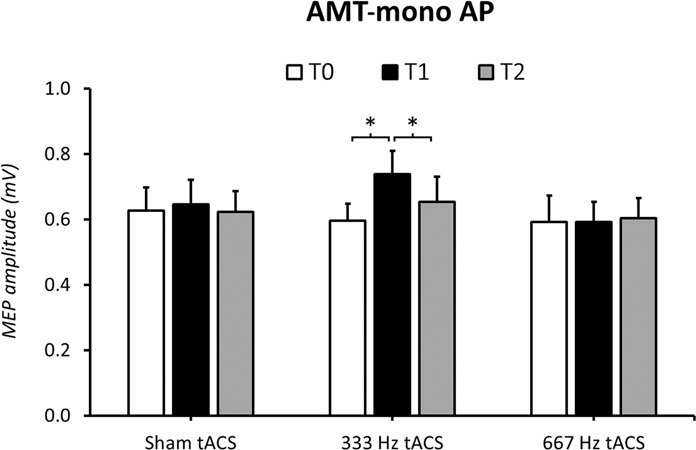
Effects of 667 Hz, 333 Hz and sham tACS in the AMT-mono AP protocol. MEPs amplitude increased during 333 Hz tACS, whereas 667 Hz tACS and sham tACS did not exert any effect. Asterisks denote significant differences in the post-hoc analyses.

The rmANOVA conducted on MEP amplitude in Experiment 2 disclosed a significant ‘session’ × ‘time-point’ interaction (F_4,56_ = 2.99, p = 0.03), suggesting frequency- and time-dependent effects of tACS. The factor ‘session’ was not significant (F_2,28_ = 0.96, p = 0.39). Separate rmANOVAs with ‘time-point’ as factor were, then, conducted for each session. The analysis indicated that 333 Hz tACS modulated AMT-mono AP protocol (F_2,28_ = 7.12, p < 0.01), while 667 Hz (F_1.4,19.2_ = 0.09, p = 0.84) and sham tACS (F_2,28_ = 0.49, p = 0.61) left it unchanged. Post-hoc analyses demonstrated that MEPs amplitude increased at T1 (T0 vs T1: p < 0.01), but not at T2 (T0 vs T2: p = 0.7; T1 vs T2: p = 0.02) (Fig. 4).

Finally, the rmANOVAs performed on MEP amplitude in the control experiment resulted in a non-significant factor ‘time-point’ both for AMT-biph (T0: 0.47 ± 0.20 mV, T1: 0.46 ± 0.24 mV, T2: 0.58 ± 0.31 mV; F_2,28_ = 1.83, p = 0.18) and AMT-circ (T0: 0.47 ± 0.21 mV, T1: 0.62 ± 0.37 mV, T2: 0.56 ± 0.42 mV; F_2,28_ = 1.22, p = 0.31), suggesting no effects of tRNS on these TMS measures of M1 excitability.

**Figure 5:**
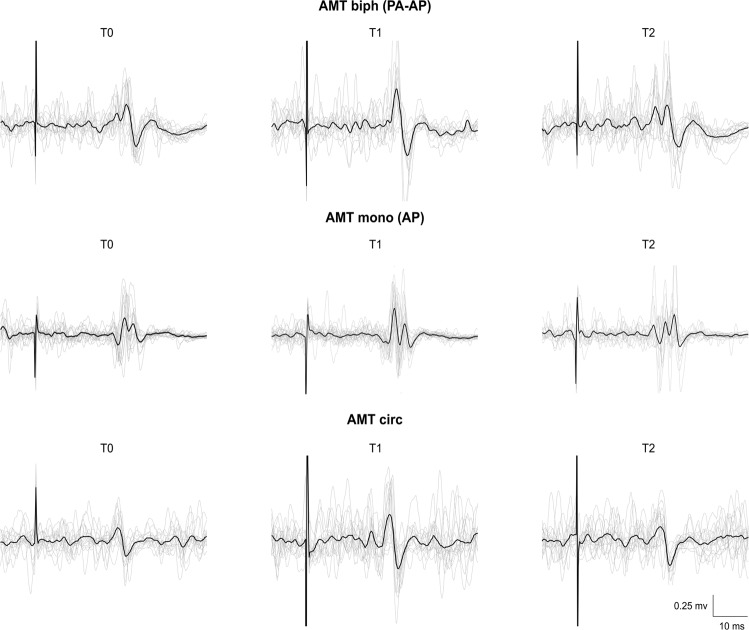
Effects of 333 Hz tACS in representative subjects. The amplitude of MEPs evoked by biphasic TMS pulses delivered during voluntary muscular contraction through a standard figure-of-eight coil at the intensity of 100% AMT (AMT-biph – upper line) and by monophasic TMS pulses delivered through a figure-of-eight coil with the handle AP-oriented (AMT-mono AP – middle line) increased during 333 Hz tACS (T1). In contrast, the amplitude of MEPs evoked by monophasic TMS pulses delivered during voluntary muscular contraction through a circular coil at the intensity of 100% AMT (AMT-circ – lower line) increased both during and 5 minutes after 333 Hz tACS over M1 (T2).

The effects of 333 Hz tACS in representative subjects are illustrated in Fig. 5.

## Discussion

In this study, we systematically investigated the effect of tACS, delivered at two different high-frequencies (i.e., 667 and 333 Hz), on the amplitude of MEPs elicited by distinct TMS protocols consisting of different current orientations across the central sulcus, stimulation intensities and muscle contraction states (i.e., rest versus active). We used TMS protocols thought to preferentially target different sites/circuits within the motor cortex: a protocol (monophasic pulses, PA-induced current at three different intensities) which indirectly activates the CSNs evoking the 667 Hz bursting; two protocols (biphasic pulses, PA-AP-induced current, at low intensity; monophasic pulses, AP-induced current, at low intensity) which indirectly activate the CSNs evoking the 333 Hz bursting; a protocol (monophasic pulses, anticlockwise current flow, at low intensity) that preferentially activates more directly the CSNs. We demonstrated that the amplitude of MEPs evoked by low-intensity biphasic as well as monophasic AP-oriented TMS stimuli during 333 Hz tACS on the motor cortex is enhanced. tACS at 333 Hz also facilitated MEPs evoked by low intensity TMS delivered through a circular coil, an effect that weakly persisted 5 minutes after the end of tACS. By contrast, 667 Hz tACS and sham tACS did not produce any significant effect on amplitude of MEPs recorded both at rest and during mild voluntary contraction. Finally, our control experiment showed that tRNS at 10–640 Hz is not able to modulate MEPs evoked by low-intensity biphasic PA-oriented TMS and monophasic TMS delivered through a circular coil.

Since motor thresholds were comparable between the three different sessions, we can exclude that different baseline levels of corticospinal excitability influenced our results. In addition, as we studied all the subjects at the same time of the day, we can assume that circadian fluctuations of M1 excitability did not impact on our data. All the TMS protocols were delivered in a random order before, during and after tACS. Also, differently from previous reports^12,18,27,28,32^, in our study tACS did not induce visual, skin or other sensations in any subject (60 applications of 333/667 Hz tACS in total). Thus, none of the participants was able to distinguish among the three different sessions. In addition, the stimulation frequencies tested in our experiments have never been used so far, and are not currently included in the safety guidelines for TES^26^. The data on the absence of side effect may be, therefore, useful to expand the safe stimulation frequency range for tACS. Sham tACS did not produce any change of MEP amplitude, so making it unlikely that attentional or placebo effects biased our results. Finally, the ad-hoc analysis conducted on EMG data recorded immediately before TMS demonstrated comparable values at T0, T1 and T2, excluding the possibility that different amount of voluntary muscular contraction influenced MEP amplitude during and after 333 Hz tACS.

Our results show that the effect of tACS is frequency-specific, since MEPs were facilitated only when we applied tACS at 333 Hz, and circuit-specific since MEPs were facilitated only with three of the TMS protocols tested. MEPs were facilitated when using both a biphasic PA-AP and a monophasic AP current flow across the central sulcus. By contrast, MEPs were unchanged when monophasic pulses were delivered using a PA-induced current. Recordings of corticospinal volleys in humans showed that both PA-AP biphasic and AP monophasic TMS evoke a descending activity, often characterized by longer latency waves and lower frequency of discharge, corresponding at about 333 Hz^5,33^. Thus, we here hypothesize that tACS, delivered at this specific frequency band, increased the MEP amplitude by reinforcing the synchronization of a cortical circuit characterized by a physiological activity at ≈333 Hz. Also, we speculate that the specific AP orientation of the current induced in the brain is particularly important for the activation of such putative 333 Hz oscillatory intracortical network. The neuronal elements are entrained by tACS because the endogenous and exogenous polarizing mechanisms are additive^17,18,22^. The effects of tACS on TMS-evoked bursting at 333 Hz might be explained by an interaction between the two forms of stimulation. AP and biphasic TMS produce a CSN bursting with a component aligned at 333 Hz together with a less synchronized corticospinal activity^5^. While weak alternating current at 333 Hz enhances the tendency of the CSNs to oscillate at this frequency, thus, there is a cooperative effect that enhances the phase alignment and the bursting at this specific frequency. This phenomenon is known as intrinsic resonance and can be induced by very low intensities of stimulation^17,20,34^. Another finding of our study is that by using low intensity monophasic TMS with a circular coil, MEP amplitude again increased during 333 Hz tACS. At low intensity, this type of stimulation is thought to preferentially activate pyramidal neurons at the axon hillock^5,30,35^, but, probably because it stimulates a large area of the brain containing neurons oriented at different angles, it can also evoke small peaks of activity that do not match the peaks of the 667 Hz bursting^35^. Thus, the CSNs are likely activated both directly and pre-synaptically when a circular coil is used. Accordingly, MEPs facilitation during 333 Hz tACS may suggest that this stimulation makes the resonant endogenous 333 Hz circuit more responsive to circular coil TMS. By using a circular coil, we also found that MEP enhancement weakly persisted for several minutes after the end of 333 Hz tACS. One possibility to explain this result is that the entrainment produced by tACS persisted after the stimulation ended. This is, however, rather unlikely since this long-lasting entrainment would have similarly increased MEPs elicited by biphasic TMS at T2, and that was not the case. In addition, none of the previous TMS-tACS studies have demonstrated after-effects of tACS on M1 excitability^13,14,28,36^, with the exception of Moliadze *et al*.^31^, who applied tACS in the ‘ripple frequency’ range. We, thus, hypothesize that high-frequency tACS may promote brain plasticity processes under specific experimental conditions. To this regard, the particular pattern of CSNs activation produced by the circular coil stimuli, implying simultaneous pre-synaptic (via cortico-cortical projections) and post-synaptic activation at the axon hillock level, would be more prone to demonstrate such after-effects.

It is interesting to note that Moliadze *et al*.^31^ observed facilitatory effects of high-frequency (140 Hz and 250 Hz) tACS on M1 excitability using PA TMS at intensities significantly above RMT (i.e. eliciting ≈1 mV MEPs). The higher frequency used by Moliadze *et al*. is relatively close to the lower frequency used in the present study that, in contrast to 250 Hz tACS, produced no effect on PA-evoked MEPs. The differential effects of 250 and 333 Hz on PA-evoked MEPs together with the differential effects of 333 Hz on PA- and AP-evoked MEPs reveal a strong frequency- and circuit-specificity of different oscillatory neurostimulation patterns. This suggests that MEP changes induced by different frequencies might build on different mechanisms, such as the engaging of some neuronal networks at their intrinsic frequency, or the selective engaging of subnetworks or functionally connected networks, or the perturbation of intrinsic oscillatory activities. The hypothesis of multiple, frequency-related mechanisms is also supported by the findings of Moliadze *et al*.^31^, showing that even though both 140 and 250 Hz were facilitatory, there was a consistent difference in the effects in terms of amount and duration of facilitation. It should also be considered that the results of the present study are not entirely comparable with those by Moliadze *et al*., who used a stimulus intensity higher than the 110% RMT intensity we applied in our study. A low TMS intensity is known to generate a short burst of high-frequency corticospinal activity, while higher intensities produce a more prolonged bursting activity due to the activation of additional intracortical circuits^5,30^. Thus, the lack of effects observed with 333 Hz tACS on PA-evoked MEPs at 110% RMT in our experiments may depend not only on the different frequency of tACS used but also on the different population of intracortical circuits activated by TMS.

**Figure 6:**
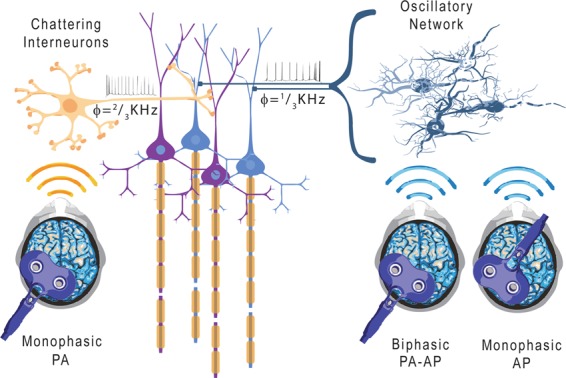
Hypothetical representation of the cortical microcircuits producing bursting activity of corticospinal cells at 667 and 333-Hz. Based on the results of this study, we speculate that the bursting of corticospinal cells is produced by two different mechanisms acting on different corticospinal cells. 1) Left: activation of chattering interneurons discharging at 667 Hz connected to the corticospinal cells. These elements are activated by monophasic magnetic stimulation with posterior-to-anterior (PA) current flowing across the central sulcus. 2) Right: activation of an oscillatory circuit with a native frequency of 333 Hz connected to the corticospinal cells. These elements are supposed to be activated by biphasic magnetic stimulation with posterior-to-anterior followed by anterior-to-posterior (PA-AP) current flowing across the central sulcus and by monophasic magnetic stimulation with anterior-to-posterior (AP) current flowing across the central sulcus. This oscillatory circuit is entrained by 333 Hz tACS.

The higher frequency (667 Hz) tACS did not modify MEPs amplitude. Although we cannot fully exclude detecting an effect by using significantly larger sample size (i.e. type II statistical error), we may assume that this effect, if any, would be weaker than that of the 333 Hz tACS. This might appear surprising because 667 Hz coincides with the frequency of the main bursting activity evoked by TMS. One possibility is that the circuit producing the high frequency bursting is composed of neurons with a high propensity to synchronize and tACS cannot further enhance synchronization (ceiling effect). However, even if this were the case, a facilitatory effect should be present at least at the lowest stimulus intensity that usually evokes a single descending wave^5^. Since the effects of tACS strongly depend on how the induced intracranial field relates to neural structures^22^, another possibility is that the orientation of cortical neurons that form the network discharging at higher frequency make them less responsive to the induced electric field. Different cortical neurons respond to different orientations of the induced current in the brain and this is relevant both for the excitatory and the inhibitory neurons^37^. A further possibility is that the mechanisms that produce the high-frequency bursting differ from those producing the lower-frequency ones. *In vivo* and *in vitro* recordings have identified cortical neurons capable of generating bursts of activity at more than 600 Hz. These neurons have been termed chattering cells^38^. Fast-spiking inhibitory interneurons producing a bursting activity at 600 Hz have been demonstrated in the somatosensory cortex using intracellular recordings^39,40^. In analogy, the high frequency bursting of the CSNs after PA-TMS might be produced by the activation of nearby chattering interneurons at 667 Hz, instead of being the effect of a network of oscillatory activity. If the high frequency bursting were produced by intrinsic membrane properties of cortical interneurons that respond with rapid firing to TMS and in turn activate CSNs, then the intensity of tACS used in present experiments might not suffice to influence their response because much higher fields are needed to modulate silent neurons^22,41^. The phenomenon of stochastic resonance has instead a much lower threshold when the tACS modulation interacts with a network that is spontaneously oscillating at the same frequency^22^. Thus, it might be that the presence of two CSN bursting modes is due to the activity of two different mechanisms that produce them: 1) the repetitive firing of cortical interneurons with intrinsic oscillatory properties connected to CSNs produces the 667 Hz bursting; 2) a more complex network with a native oscillatory frequency of 333 Hz probably connected to a different population of CSNs produces the bursting at this frequency (Fig. 6).

Finally, it is interesting to note that there was no effect of 667 Hz tACS on MEPs associated with 333 Hz corticospinal bursting induced by TMS and no effect of 333 Hz tACS on MEPs associated with 667 Hz bursting. This suggests that the circuits may be independent and that the CSNs targeted by the two sources of inputs do not overlap. This is in agreement with the intra-axonal recordings of Maier *et al*.^6^ who showed different corticospinal axons discharging at different frequencies after intracortical electrical stimulation, suggesting that different CSNs respond at a different frequency to the same stimulus.

The main limitation of the study is that our hypotheses are based on indirect evidence of ‘resonance’. Indeed, due to technical limitations related to the presence of the electrical artifact, it is impossible to record the EEG activity during tACS and provide direct proof of 333 Hz activity increase during the stimulation.

Concluding, we here provide the first evidence that specific neuronal elements connected to the CSNs are resonant to 333 Hz tACS. We also show that elements making CSNs bursting at 667 Hz, are resonant neither to 667 Hz nor to 333 Hz tACS. These findings suggest that there are at least two independent human motor cortex circuits evoking corticospinal activity at different frequencies. Using non-invasive brain stimulation techniques these circuits can be targeted selectively and their output can be modulated for the lower frequency band of activity. A better knowledge of the cortical circuits producing corticospinal outputs, and the development of protocols for selective evaluation and modulation of these circuits, might be useful to clarify the pathophysiological basis of motor disorders and, also, for the development of neuromodulation approaches aimed at restoring the physiological corticospinal output in these conditions.
